# Biomaterials and tissue engineering strategies for posterior lamellar eyelid reconstruction: Replacement or regeneration?

**DOI:** 10.1002/btm2.10497

**Published:** 2023-02-15

**Authors:** Yuxin Yan, Qiumei Ji, Rao Fu, Chuanqi Liu, Jing Yang, Xiya Yin, Qingfeng Li, Ru‐Lin Huang

**Affiliations:** ^1^ Department of Plastic and Reconstructive Surgery Shanghai Ninth People's Hospital, Shanghai Jiao Tong University School of Medicine Shanghai China; ^2^ Department of Plastic and Burn Surgery West China Hospital, Sichuan University Chengdu China

**Keywords:** conjunctiva, eyelid reconstruction, posterior lamella, regenerative medicine, tarsal plate, tissue engineering

## Abstract

Reconstruction of posterior lamellar eyelids remains challenging due to their delicate structure, highly specialized function, and cosmetic concerns. Current clinically available techniques for posterior lamellar reconstruction mainly focus on reconstructing the contour of the eyelids. However, the posterior lamella not only provides structural support for the eyelid but also offers a smooth mucosal surface to facilitate globe movement and secrete lipids to maintain ocular surface homeostasis. Bioengineered posterior lamellar substitutes developed via acellular or cellular approaches have shown promise as alternatives to current therapies and encouraging outcomes in animal studies and clinical conditions. Here, we provide a brief reference on the current application of autografts, biomaterials, and tissue‐engineered substitutes for posterior lamellar eyelid reconstruction. We also shed light on future challenges and directions for eyelid regeneration strategies and offer perspectives on transitioning replacement strategies to regeneration strategies for eyelid reconstruction in the future.

## 1 INTRODUCTION

**FIGURE 1:**
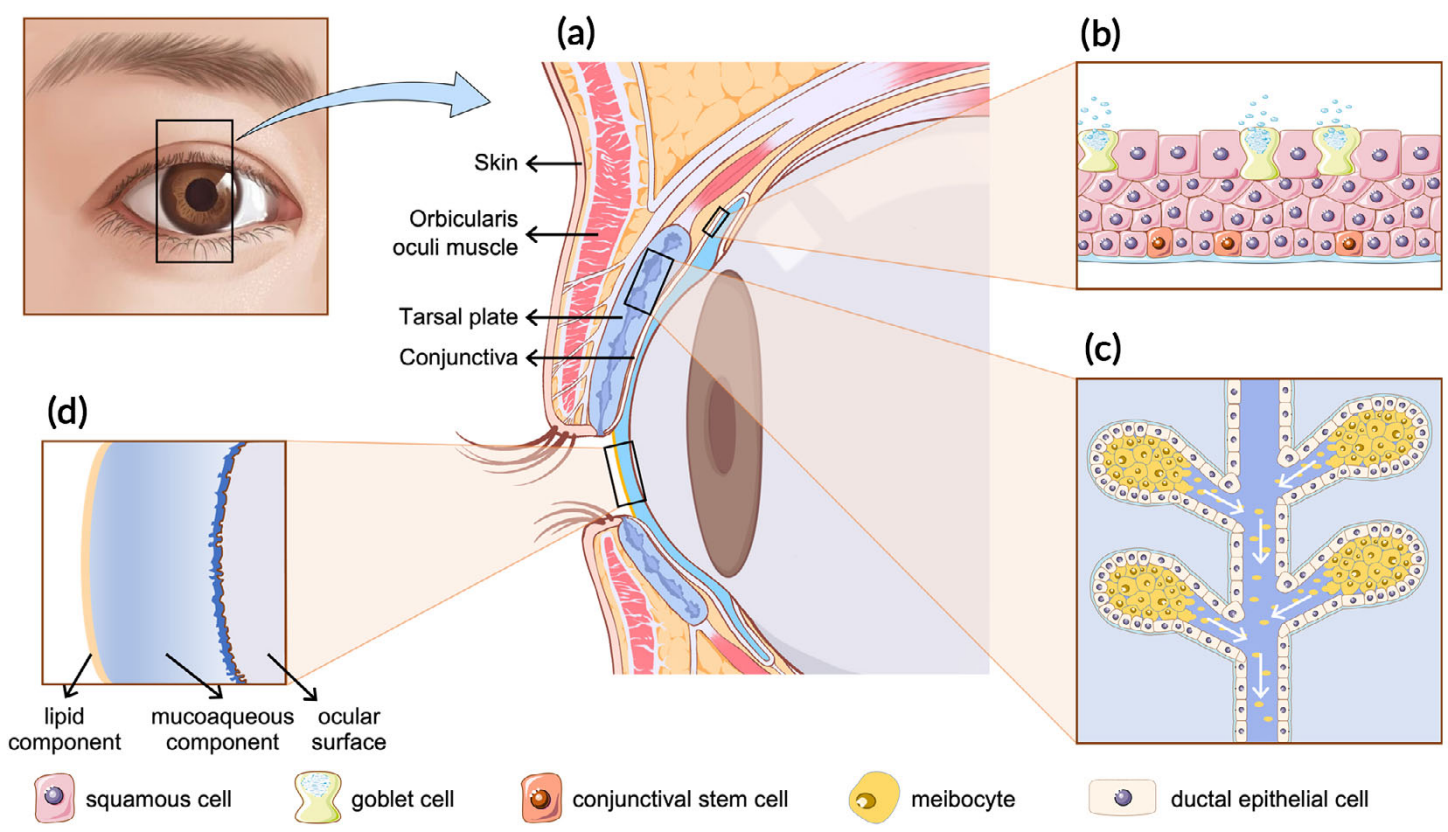
Illustration of the anatomical structure of the eyelid. (a) Eyelid. (b) Conjuctiva. (c) Meibomian gland. (d) Tear flim.

**FIGURE 2:**
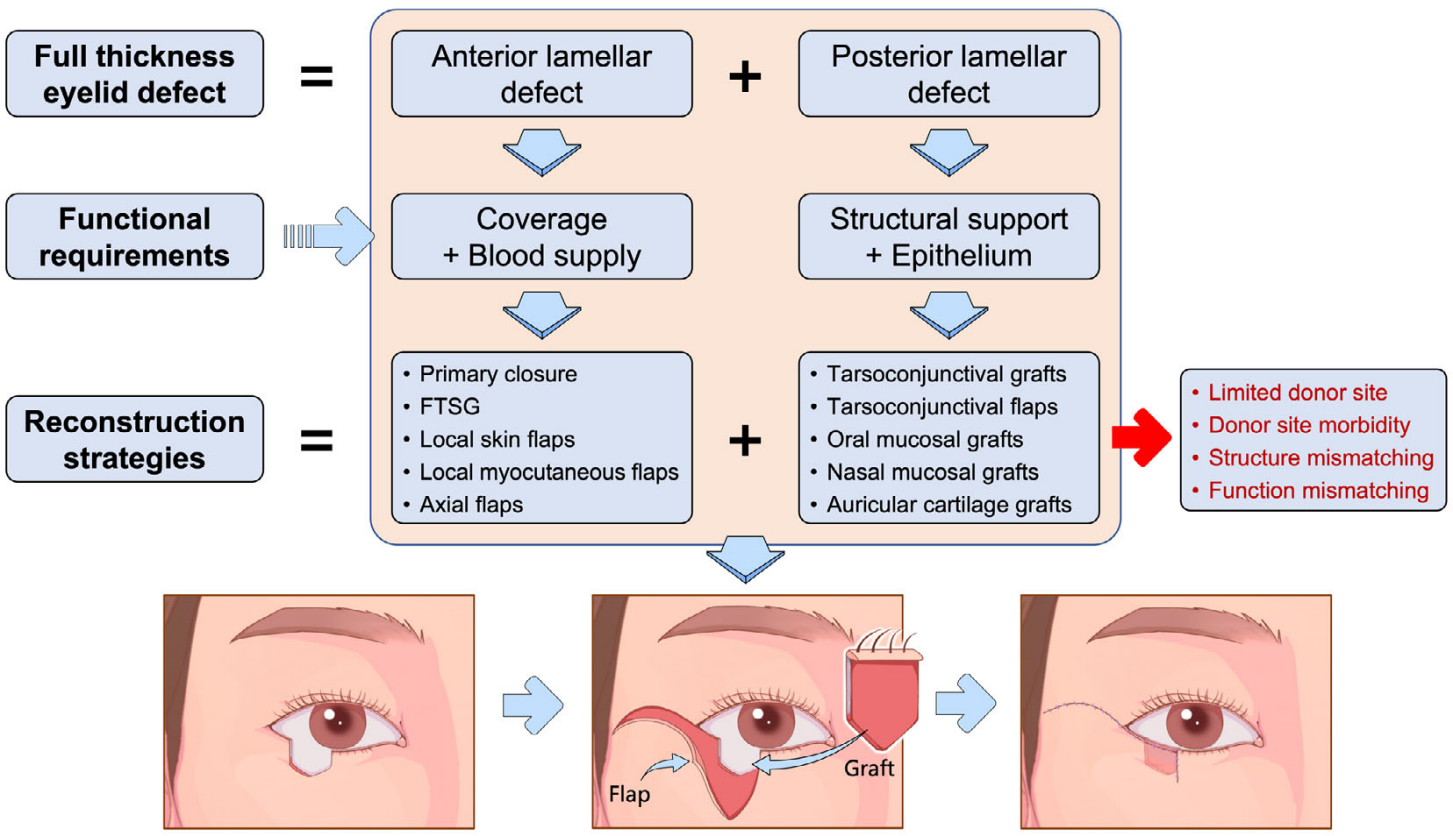
Illustration of the surgical strategies of eyelid reconstruction. FTSG, full thickness skin graft.

The eyelids play critical roles in protecting the globe, maintaining ocular surface hydration, and expressing emotions. Anatomically, the eyelids contain two layers, namely, the anterior lamella and the posterior lamella^1^ (Figure 1a). The eyelids not only physically provide protective coverage for the cornea and the globe but also serve important roles in maintaining ocular health and preserving visual integrity. Esthetically, the frame of the eye is distinctively defined by the position and shape of the eyelids. Thus, eyelid reconstruction must reconstruct both structure and function of the eyelids and restore an esthetically acceptable appearance with minimal surgical morbidities. To achieve these goals, we previously proposed that eyelid defects should be reconstructed following the “like for like” reconstructive principle and the surgeon should take into consideration the characteristics of the defects, including the thickness, size, and location.^2^ By following these principles, numerous well‐developed surgical techniques, including free skin grafts and tissue flaps, have been applied to repair skin or myocutaneous defects in anterior lamellar reconstruction, with satisfying functional and cosmetic outcomes in clinical practice.^3^, ^4^, ^5^ However, due to the unique structural and functional properties of the eyelid, as well as the lack of histologically ideal donor tissues, current clinically available surgical techniques have shown limited clinical success in posterior lamellar reconstruction^6^, ^7^, ^8^ (Figure 2).

The current strategies for posterior lamellar reconstruction mainly depend on the defect size. In general, small defects involving less than 25% of the eyelid width are preferably repaired by direct closure. Moderate defects involving 25%–50% of the eyelid width can also be reconstructed by primary closure in combination with cantholysis or local tarsoconjunctival flap transfer. For the defects involving at least 50% of the eyelid width, free composite autografts, Hughes tarsoconjunctival flaps, Tenzel semicircular flaps, tarsal transposition flaps, or combinational techniques are usually required to provide primary coverage and additional lubrication for the globe.^1^, ^2^, ^9^ However, these reconstructive techniques are aimed at replacing the defective tissue with similar autologous tissue to restore the contour of the eyelid. And there is no treatment option capable of restoring the natural function of the eyelid. The development of restorative, instead of palliative, management strategies for eyelid defects in terms of both function and structure requires extensive surgical knowledge and versatility; thus, there is interest in tissue engineering‐based therapies.

**FIGURE 3:**
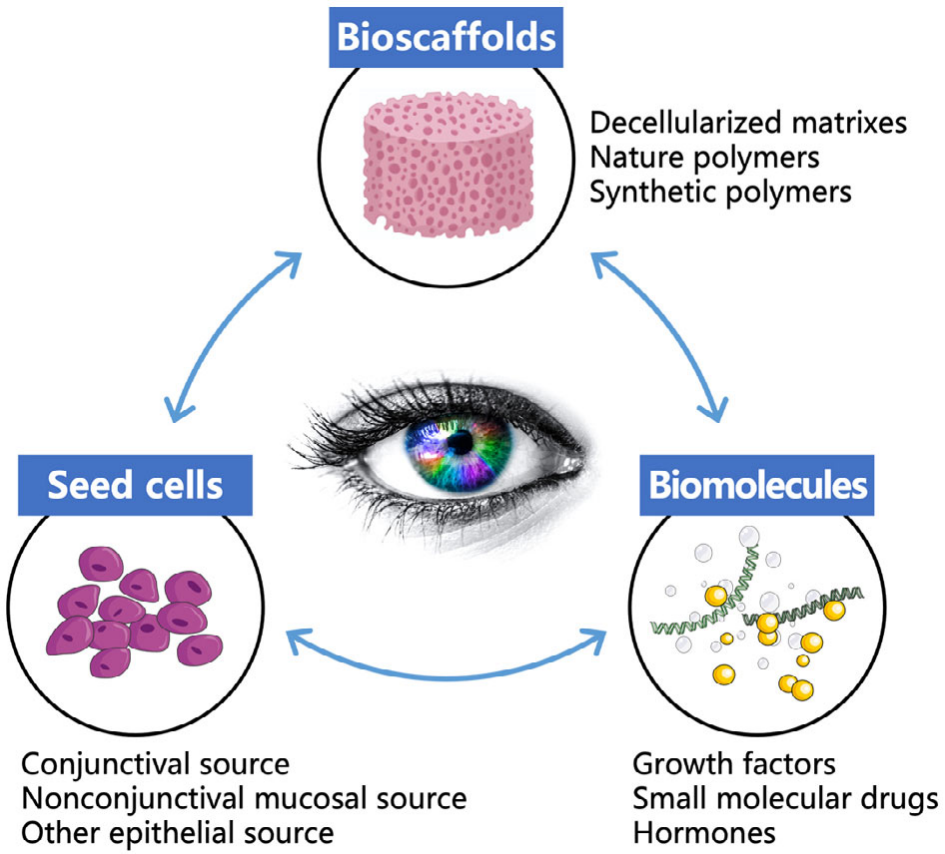
The three basic elements in eyelid tissue engineering.

**FIGURE 4:**
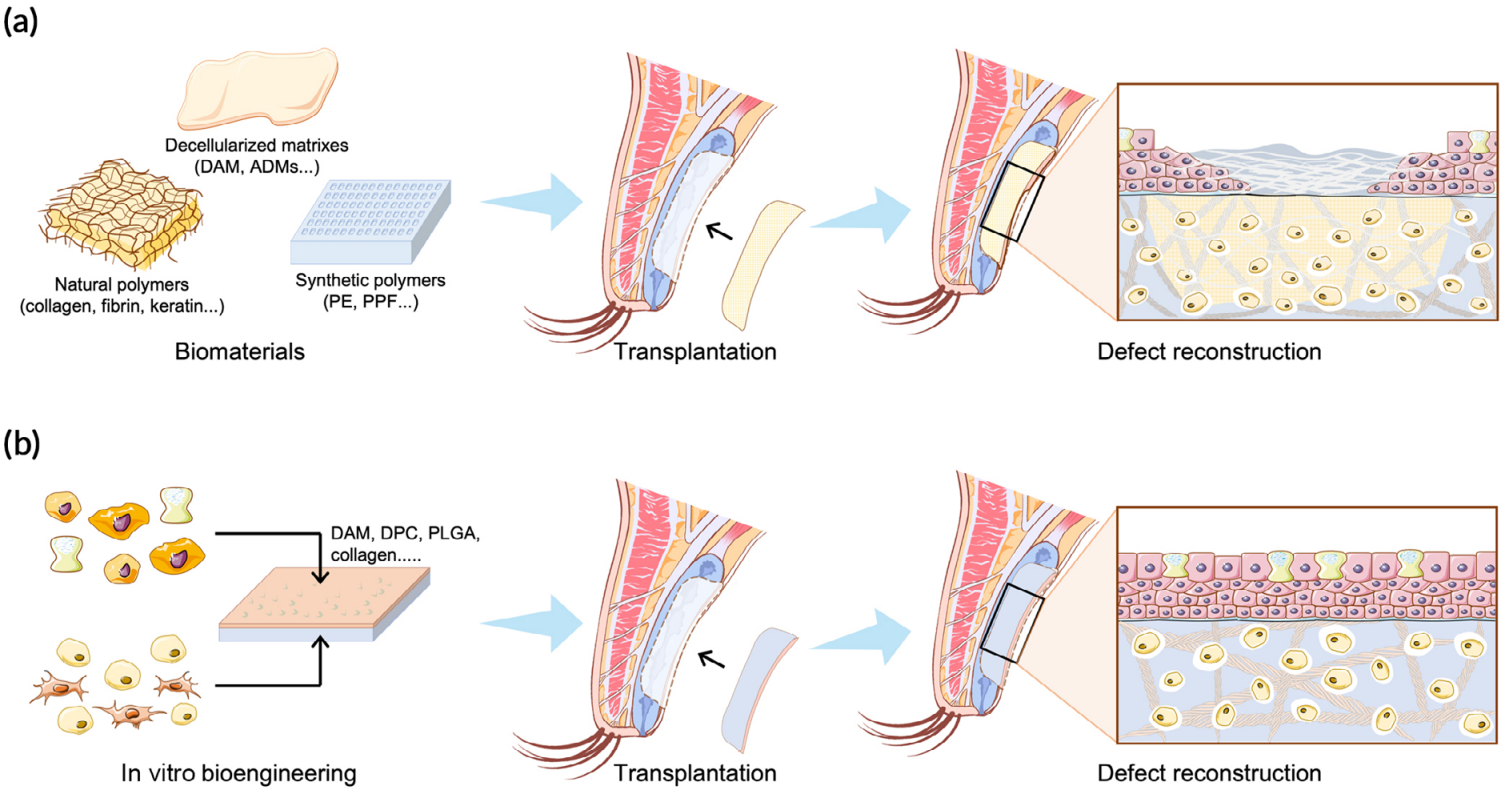
Schematic diagram of acellular and cellular approaches for posterior lamellar reconstruction. (a) Acellular approach. (b) Cellular approach. ADMs, acellular dermal matrixes; DAM, decellularized amniotic membrane; PDC, porcine decellularized conjunctiva; PE, porous polyethylene; PLGA, poly(lactic‐*co*‐glycolic) acid; PPF, poly(propylene fumarate).

Bioengineering represents the future of reconstructive surgery and is emerging as a promising alternative for eyelid reconstruction (Figure 3). Ideally, bioengineered posterior lamellar substitutes should restore key structures and functions of the eyelid without additional donor‐site morbidities or postoperative complications. To date, many innovative native tissue grafts,^10^, ^11^ biomaterials,^12^, ^13^ and bioengineered tissues^14^, ^15^ have been proposed as posterior lamellar substitutes, some of which have been clinically used for eyelid reconstruction and have yielded promising preliminary results.^11^, ^14^, ^15^ The bioengineering of posterior lamellar substitutes can be categorized into two distinct approaches: (1) acellular approaches, in which natural or synthetic acellular biomaterials are transplanted into defect areas to act as bioscaffolds for guiding tissue regeneration in vivo by leveraging endogenous cells and microenvironments (Figure 4a); and (2) cellular approaches, in which bioscaffolds are precellularized in vitro to mimic specific functions of the target tissue and then transplanted to promote tissue regeneration (Figure 4b). It appears that the presence of functional cells within the bioscaffold before implantation is particularly important for successful eyelid reconstruction, and strategies based on cellular approaches have indeed achieved promising results in preclinical studies.^16^, ^17^, ^18^ However, these laboratory technologies are still in the progress of translation from bench to bedside, which will face many challenges. In this review, the eyelid anatomy will be briefly presented first to provide context for the requirements of ideal posterior lamellar substitutes, followed by an overview of current biomaterials and tissue engineering strategies for posterior lamellar eyelid reconstruction. Finally, the ongoing challenges and future directions of eyelid regeneration strategies will be discussed, and some perspectives will be offered on transitioning replacement strategies to regeneration strategies for eyelid reconstruction in the future.

## 2 EYELID ANATOMY: FOCUSING ON THE POSTERIOR LAMELLAR

The eyelid is essentially a bilamellar structure that composed of the anterior and posterior lamella (Figure 1a). The former consists of the skin and the orbicularis oculi muscle, providing blood supply to the lamellar structures. The eyelid skin is thin and lacks subcutaneous fat. It is highly elastic and able to facilitate eyelid movement. The orbicularis oculi muscle, consisting of the preseptal, orbital, and pretarsal subunits, is responsible for eyelid closure. By using the laxity of adjacent tissues, local skin or myocutaneous flaps are used to reconstruct skin and muscle defects of the anterior lamella.^2^

The posterior lamellar comprises the palpebral conjunctiva and the tarsal plate, which form histological foundations of the two primary functions of the posterior lamellar, that is, structural support and corneal protection via a mucosal surface. The tarsal plate is a semilunar‐shaped structure approximately 25 mm in length and 1 mm in thickness, while the height of the tarsal plate ranges from 8 to 12 mm in the upper eyelid and 4 to 5 mm in the lower eyelid.^19^, ^20^ The tarsal plate is a distinct transitional connective tissue which possesses the characteristics of both cartilage and dense fibrous connective tissue.^21^, ^22^ Histologically, the tarsal plate is not a truly fibrocartilaginous structure^23^, ^24^ but consists mainly of abundant meibomian glands secreting meibum and extracellular matrix (ECM) surrounding the fibroblasts. The ECM of the tarsal plate mainly contains collagen (Col) I, Col III, Col IV, aggrecan, and tenascin, along with various glycosaminoglycans. The interactions between collagen and other ECM components contribute to the rigidity and mechanical strength of the eyelid.^24^, ^25^, ^26^ The meibomian glands are individual branched alveolar sebaceous glands consisting of duct systems and secretory acini. The ducts extend to the entire length of the tarsal plate and open at the free margin of the eyelid. Histologically, a meibomian gland consists of stratified squamous keratinized ductal epithelial cells and meibocytes arranged as functional secretory units called acini^27^ (Figure 1c). The meibum, an essential composition of the tear film, is spread out when meibomian gland ducts are compressed by the transverse muscle fibers during blinking. Meibomian gland dysfunctions can result in ocular and eyelid discomfort, ocular surface diseases such as evaporative dry eye.^28^

The conjunctiva, which arises from the corneoscleral limbus and overlies the inner surface of the eyelid, comprises a nonkeratinized stratified columnar and stratified squamous epithelium interspersed with goblet cells and a vascularized basement membrane composed of laminin and Col IV.^29^ The epithelium is composed of stratified squamous nongoblet cells (90%–95%), goblet cells (5%–10%), and occasional lymphocytes and melanocytes^30^ (Figure 1b). Soluble mucins can be secreted by the goblet cells, which serve as a crucial component of the tear film to bathe the ocular surface (Figure 1d). Conjunctival loss or scarring that can result from conjunctival damage or ocular surface disease may lead to eyelid misalignment, limited eye movement, diplopia, and dry eye symptoms.^31^ In addition, it should be noted that the conjunctiva can spontaneously re‐epithelialize upon injury, as both stratified squamous nongoblet and goblet cells can be regenerated continuously by conjunctival stem cells,^32^ inspiring tissue engineering strategies using conjunctival stem cells to repair conjunctival defects.

Collectively, the posterior lamella is a delicate bilamellar structure that serves two primary functions: mechanical support for the eyelid and sufficient lubrication of the cornea. Specifically, the tarsal plate provides mechanical support, maintains the appearance of the eyelid, and prevents abnormalities such as corneal exposure and eyelid retraction, making it an essential part of physical appearance and eyelid functions. The conjunctiva provides a mucosal surface that allows smooth globe movement; additionally, the goblet cells within the conjunctiva and the meibomian glands within the tarsal plate secrete mucins and lipids, respectively, which stabilize the tear film to maintain ocular surface homeostasis. To achieve functional eyelid reconstruction, an ideal posterior lamellar substitute should have the following characteristics: (1) mechanical strength: the posterior lamellar substitute should be a thin, elastic and stable construct that possesses considerable mechanical strength to maintain a semilunar shape, match the contour of the globe, and stabilize eyelid movement; (2) a smooth epithelialized inner surface: the construct should support efficient epithelial repopulation and stratification to mimic the conjunctiva; and (3) secretory functions: the substitute should produce sebaceous and/or mucin‐like substances to stabilize the tear film and prevent dry eye.

## 3 AUTOLOGOUS TISSUE GRAFTS

### 3.1 Tarsoconjunctival, tarsal, and tarsomarginal grafts

**FIGURE 5:**
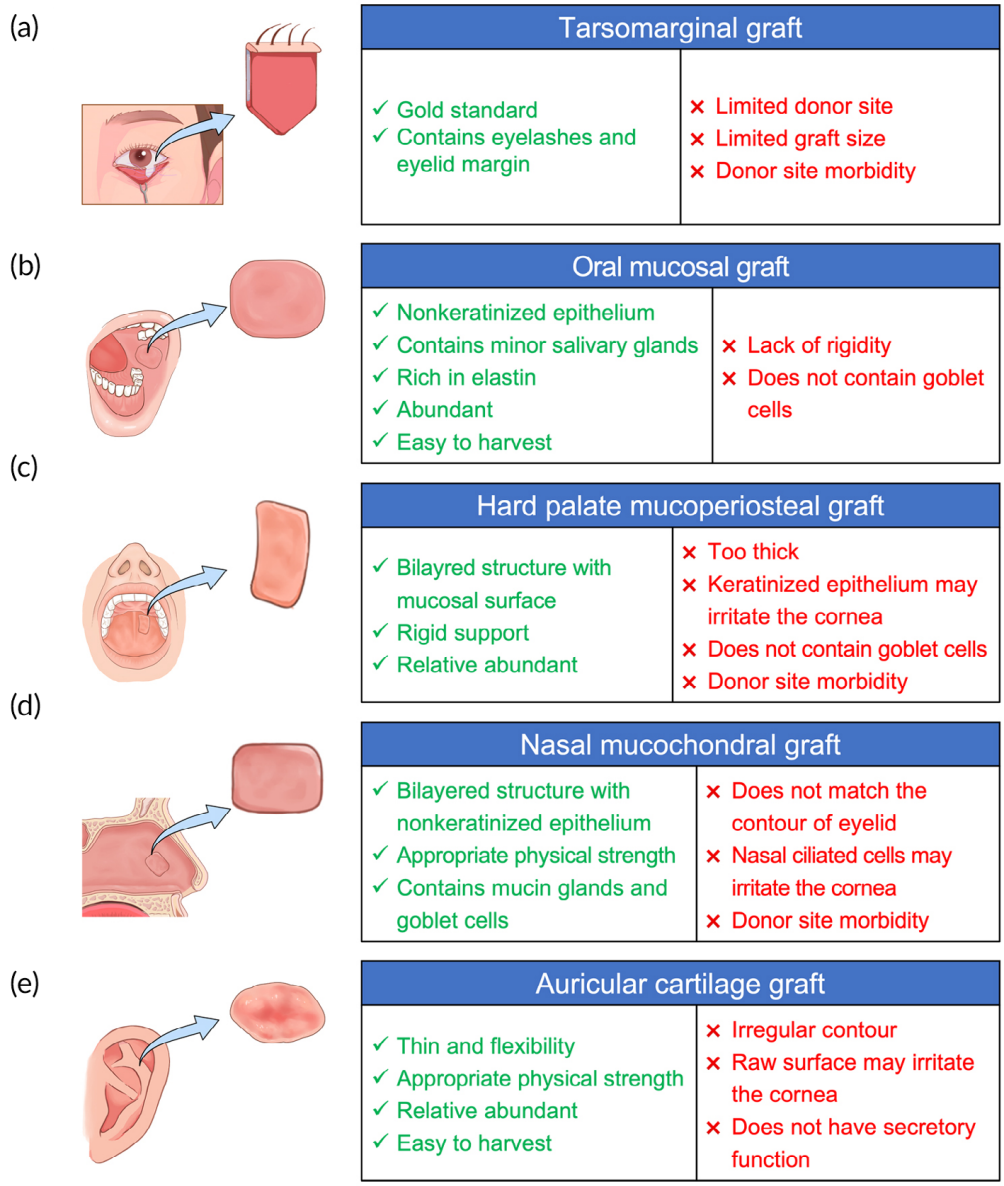
Summary of different autografts for posterior lamella eyelidreconstruction. (a) Tarsomarginal graft. (b) Oral mucosal graft. (c) Hard palate mucoperiosteal graft. (d) Nasal mucochondral graft. (e) Auricular cartilage graft.

Autografts that could provide physical support and/or a mucosal layer have been applied in clinical practice for posterior lamella reconstruction (Figure 5). According to the three main features of the posterior lamella, the tarsoconjunctival graft is the golden standard for posterior lamella reconstruction and can be used to reconstruct the defects involving less than 75% of the whole eyelid width.^33^, ^34^, ^35^ Compared with tarsoconjunctival flaps, such as the Hughes flap,^36^, ^37^ Cutler‐Beard flap,^38^, ^39^ and Mustarde lower lid switch flap,^40^, ^41^ free tarsoconjunctival grafts can reconstruct the defect eyelid in a single‐stage surgery, which is particularly suitable for elderly patients after malignant eyelid tumor resection. Tarsal grafts can provide posterior lamellar replacement without the conjunctival lining. When used for repairing full‐thickness defects, free tarsoconjunctival or tarsal grafts are often used in combination with other vascularized flaps.^42^, ^43^ As the free tarsal graft lacks conjunctival lining, it is usually left alone to allow gradual re‐epithelialization through the migration of the marginal conjunctiva.

Tarsomarginal graft is a composite graft, which is composed of the tarsal plate, conjunctiva, lid margin, and eyelashes (Figure 5a). Currently, tarsomarginal grafts of One‐fourth and even one‐third of the whole eyelid width can be obtained by primary closure.^44^ Tarsomarginal grafts are suitable for eyelid defect reconstruction in various areas, including medial, central, or lateral defects in both lower and upper eyelids, particularly, the defects involving the eyelid margin, as these grafts preserve the native structures of the lid margin. As a free graft, the tarsomarginal graft should be covered with a local myocutaneous flap or a distal axial flap to ensure sufficient blood supply, which allows simultaneous transplantation of not only one but also two even three tarsomarginal grafts harvested from different donor eyelids. The main limitations of these autologous grafts include small graft size, eyelid retraction, scar formation, and donor eyelid morbidity,^45^ which hinder their clinical application.^44^, ^45^ The postoperative complication rate for free tarsoconjunctival graft implantation has been reported to be as high as 43% in the East Asian population^35^ and 84% in the Caucasian population.^46^ Ectropion and entropion are the most common complications in the lower and upper eyelids, respectively.

### 3.2 Nontarsoconjunctival grafts

To overcome the constraints of posterior lamella‐derived autografts, mucous membrane autografts harvested from other areas, including the lip,^47^, ^48^ buccal mucosa,^49^, ^50^ gingival alveolar mucosa,^51^ hard palate,^52^, ^53^ and nasal septum,^54^, ^55^ have been developed as replacements.

#### 3.2.1 Oral mucosa grafts

The oral mucosa consists of vascular connective tissue named the lamina propria and nonkeratinized stratified squamous avascular epithelium^56^ (Figure 5b). Oral mucosal grafts obtained from different parts of the oral cavity have variable features. Oral mucosal grafts from the lip and bucca are rich in elastin, which confers resistance to shearing and compression, and are highly vascularized, which facilitates integration of the graft once transplanted. Thus, buccal and labial mucosal grafts are typically used to replace the conjunctiva or as a lining for other tarsal substitutes,^57^ such as auricular cartilage grafts^49^, ^58^ and amniotic membrane grafts.^59^, ^60^ Oral mucosal grafts can be harvested in forms of oral mucosal membrane grafts and minor salivary gland grafts, the latter one has been used to treat severe dry eye disease. The mucosal membrane can serve as a bioscaffold to support epithelial cells proliferation and growth. In addition, as shown by in vitro studies, epithelial stem/progenitor cells that exist in oral mucosal grafts can re‐epithelialize the tarsal plate and stabilize the ocular surfaces.^61^, ^62^ However, the mucous membranes do not contain goblet cells, which may lead to corneal irritation and dry eye symptoms.^63^ Hence, simultaneous minor salivary gland grafting may be warranted to solve this problem.^64^

#### 3.2.2 Hard palate mucoperiosteal grafts

The hard palate mucosa that composed of a mucosal surface and a dense fibrous connective tissue layer has a histological structure similar to that of the posterior lamella (Figure 5c). Hard palate mucoperiosteal grafts, taken from the center of the hard palate, can provide both rigid structural support and a moist mucosal surface for the eyelid, making them an optimal substitute for the reconstruction of the posterior lamella.^53^, ^65^, ^66^ However, the hard palate mucosa is a firm tissue featuring keratinized stratified squamous epithelium. Furthermore, long‐term observation revealed that the persistence of orthokeratosis and/or parakeratosis may last for years following transplantation, which may result in irritation of the cornea and cause pain.^67^ Additionally, the donor area of hard palate mucoperiosteal grafts is usually left alone for secondary healing, which may cause possible hemorrhage, donor‐site discomfort, and difficulties in eating. The gingival mucosa is a firm, thick, and keratinized mucosa that extends to the nonkeratinized alveolar mucosa, which is relatively loose and mobile. Grafts harvested from the gingival alveolar region consist of a segment of gingival mucosa 2 mm in height, with the remainder comprising alveolar mucosa. This transitional structure allows gingival alveolar mucosa grafts to be suitable for repairing posterior lamellar defects that involve the eyelid margin. The marginal palpebral area can be replaced by the tight gingival pyart of the graft to achieve a rigid and stable structure, and the nonkeratinized alveolar part can ensure a nonirritating posterior lamellar surface.^51^

#### 3.2.3 Nasal mucosal grafts

Nasal mucosal grafts harvested from the nasal septum,^68^, ^69^ turbinate,^67^ or ala^55^ bear a close histological resemblance to the tarsoconjunctiva. Furthermore, the nasal mucosa contains not only a large number of goblet cells but also subepithelial mucin glands; therefore, it is able to provide substitute mucin.^67^ Through a tight connection to the eyelid, septal mucochondral grafts can provide firmness as a tarsal substitute and a stable eyelid margin (Figure 5d). After thinning of the cartilage, grafts will curl up toward the mucosa side, matching the contour of the tarsal plate and achieving satisfying eyelid support and cosmetic outcomes.^70^ Nasal mucosal grafts harvested from the inner part of the nasal ala can avoid the risk of septal perforation and hemorrhage.^55^ In addition, composite mucosal grafts should be harvested from beyond the nasal hair area because conjunctival or corneal irritation may be caused by any remaining nasal hair.^71^

#### 3.2.4 Auricular cartilage grafts

Auricular cartilage grafts are thin elastic cartilage with suitable flexibility and appropriate physical strength (Figure 5e). Due to their lower metabolic rate and fewer vascular requirements, auricular cartilage grafts remain viable for many years after implantation. Thus, reconstruction of the posterior lamella with auricular cartilage grafts leads to a cosmetic contour and adequate support without significant graft absorption or shrinkage.^72^, ^73^ When auricular cartilage grafts are used for tarsoconjunctiva replacement, the conjunctival surface is missing and usually left for reepithelization, but the rough surface of the grafts may result in corneal irritation.^74^ Adding an oral mucosal graft for coverage could help solve this problem.^49^, ^58^

Although various autografts have been introduced for clinical posterior lamellar reconstruction, the lack of donor area, risk of disease exacerbation, donor‐site morbidity, and incomplete functional matching have hindered the clinical application of autologous tissues and have stimulated the exploration of appropriate acellular or cellular bioengineered substitutes for posterior lamellar reconstruction.

## 4 ACELLULAR APPROACHES

Acellular approaches aim to repair defective eyelid tissue and restore its key functions with natural or synthetic biomaterials. The ideal biomaterial for posterior lamellar repair should be a thin and stable matrix with high biocompatibility and have the ability to be integrated into the peripheral tarsus without provoking inflammatory responses and, importantly, should mimic the biological functions and physical structures of the natural ECM to support cell survival, proliferation, and growth. Currently, three major types of biomaterials are used for posterior lamellar reconstruction: decellularized ECM, natural polymers, and synthetic polymers.

### 4.1 Decellularized matrixes

Studies have demonstrated that the amniotic membrane can support epithelialization by offering a basement membrane and an avascular stromal matrix similar to the native conjunctiva. Furthermore, amniotic membrane possesses anti‐angiogenic, anti‐inflammatory, and anti‐scarring properties, making it the most widespread substitute for conjunctival replacement.^75^, ^76^ Clinically, decellularized amniotic membrane grafts are placed directly over the bare tarsus up to the eyelid margin^77^, ^78^ or applied in combination with other techniques for posterior lamellar reconstruction.^79^, ^80^ As a scaffold, the implanted decellularized amniotic membrane can support the growth of surrounding conjunctiva‐derived cells, including conjunctival nongoblet epithelial cells and goblet cells,^78^, ^81^ and is then absorbed within 6 months without scarring.^82^ Derived materials, such as freeze‐dried amniotic membrane,^75^ air‐dried dehydrated amniotic membrane,^83^ and urea‐de‐epithelialized amniotic membrane,^84^ have been developed to optimize the processes of amniotic membrane sterilization and storage and to better preserve the basement membrane structures, growth factor contents, and stroma matrix components. Although good functional and esthetic outcomes have been reported, the limited availability, variable standards for preparation, inconsistent tissue properties, and risk of infectious disease transmission continue to make the use of decellularized amniotic grafts problematic.^84^

In addition to decellularized amniotic membranes, decellularized porcine and human conjunctival grafts have also been tested for conjunctival defect reconstruction in animal experiments^85^, ^86^ and clinical studies.^85^ In clinical cases, transplanted decellularized porcine conjunctival grafts were integrated with autologous conjunctiva without graft disintegration or fibroplasia and were completely epithelialized at 2 weeks.^85^ In addition, other decellularized biomembranes, such as decellularized bovine or porcine pericardium^10^, ^87^ and decellularized adipose‐derived mesenchymal stromal cell matrix,^88^ have also shown promising results in facilitating the adhesion of goblet cells and conjunctival epithelial cells in conjunctival substitute engineering.

Acellular dermal matrixes originated from human (AlloDerm®, BellaDerm®),^89^, ^90^, ^91^ porcine (Endurage®),^11^, ^92^, ^93^, ^94^ and bovine (Surgimen®)^94^, ^95^ dermis provide off‐the‐shelf materials to replace the tarsal plate because of relatively low immunogenicity and good histocompatibility.^96^ Structurally, the ADM is a sturdy but flexible flat sheet comprising cross‐linked collagen matrix with a dermal surface and a basement membrane surface. The crosslinking process makes the graft resistant to absorption and decomposition, while its flexibility facilitates it to conform to ocular surface contours, and the collagen matrix provides a natural surface for epithelial migration and the infiltration of fibroblasts.^97^, ^98^ The efficiency and safety profiles of human, porcine, or bovine acellular dermal matrixes applied as lower eyelid spacer grafts have been well demonstrated in a series of level II and III clinical studies.^14^, ^89^, ^90^, ^94^ These acellular dermal matrix grafts can be inserted below the tarsal plate as a spacer to increase eyelid height and provide structural support after releasing the lower eyelid retractors^7^ and have shown outcomes in augmenting the height of retracted lower eyelids comparable to those of autologous auricular cartilage grafts and hard palate grafts.^94^ In clinical scenarios, tarsal plate defects are often accompanied by conjunctival deficiency, and reconstruction of tarsal plate defects with acellular dermal matrix grafts often requires a local conjunctival flap or other autografts for reconstruction of the inner layer lining.^11^, ^99^, ^100^ For patients with insufficient conjunctival tissue, an acellular dermal matrix graft can be transplanted to the defective posterior lamella with the dermal surface oriented toward the wound bed and the basement membrane facing the ocular surface, which allows full conjunctival epithelialization over the graft surface within 3–6 weeks postoperatively.^101^ However, the main drawback limiting the widespread use of ADM grafts is graft contraction and resorption, especially for grafts originating from humans.^102^ The mean graft contraction rate has been reported to be 57% for acellular dermal matrix grafts versus 16% for hard palate mucosal grafts.^103^

Another clinically available acellular membrane for tarsal plate reconstruction is TarSys®, which is decellularized porcine small intestinal submucosa containing Col I, Col III, Col IV, and associated glycosaminoglycans.^104^, ^105^ However, as TarSys® is a xenogeneic product, concerns due to the risk of infectious disease transmission and reported immunogenic inflammatory‐related complications remain.^106^, ^107^, ^108^

### 4.2 Natural polymers

Natural polymer scaffolds sourced from ECM components have been widely used for conjunctival defect repair due to their high degree of biocompatibility; such scaffolds include Col I,^109^ chitosan,^110^ and keratin.^111^ Among them, Col I, the most abundant protein in the conjunctival matrix, is the most commonly used. It has been demonstrated to successfully support not only conjunctival epithelial cell proliferation but also differentiation in vitro.^112^ In vivo, collagen‐based substitutes have been shown to promote conjunctival regeneration with complete epithelization, minor fibrosis, and minimal wound contracture.^109^, ^113^ However, untreated collagen hydrogels are opaque and exhibit poor mechanical stability because they are made of loosely packed collagen fibers and thus differ from the native conjunctival stroma, which limits their surgical applicability. Therefore, plastic compressed collagen gels^109^, ^112^ and vitrified collagen membranes^113^ have been developed to simulate collagen fibril arrangement in normal conjunctiva and provide enhanced mechanical strength. In vitro studies have demonstrated that plastic compressed collagen gels and vitrified collagen membranes support conjunctival epithelial cell proliferation and phenotype development.^112^, ^113^ Once implanted into the conjunctival defect area in rabbit models, these conjunctival equivalents engineered from both collagen‐based materials promoted conjunctival regeneration by supporting rapid re‐epithelization as well as sufficient goblet cell repopulation while minimizing wound contracture and fibrosis.^109^, ^113^ Other natural ECM proteins, such as gelatin,^114^, ^115^ elastin,^116^ and their combinations,^81^ have also been tested in the context of conjunctival equivalent engineering. Despite their high biocompatibility, natural polymers are still unable to completely mimic the components of the conjunctival ECM and have unpredictable degradation rates in vivo, which leads to greater uncertainty in their reconstructive effects.^117^

### 4.3 Synthetic polymers

Synthetic polymeric materials have certain physical geometries, chemical compositions, and biological properties; they are produced following standardized manufacturing processes and do not require decellularization procedures. Synthetic polymeric materials for conjunctival reconstruction should have the following properties: (1) processability into thin films with a controlled thickness; (2) biocompatibility to support cell adhesion, proliferation, and differentiation; and (3) biodegradability after implantation. A variety of synthetic polymers, including poly(acrylic acid) (PAA),^116^ poly(ε‐caprolactone) (PCL),^116^, ^118^, ^119^ poly(lactic acid) (PLA),^12^ poly(l‐lactic acid‐*co*‐ε‐caprolactone) (PLCL),^120^ and poly(vinyl alcohol) (PVA),^116^ have been tested in conjunctival repair studies. Among them, scaffolds engineered from PCL,^121^ PLA,^12^ and their derivatives^122^ have been used in conjunctival repair and have been shown to support goblet cell growth in animal models. The United States Food and Drug Administration has already approved the clinical application of PCL. Therefore, various modified PCL scaffolds have attracted intensive attention in conjunctival equivalent engineering due to their great potential for clinical translation.^116^

Additionally, synthetic polymers, such as porous polyethylene (PE),^13^, ^104^, ^123^ poly(3‐hydroxybutyrate‐*co*‐3‐hydroxyhexanoate) (PHBHHx),^124^ poly(propylene fumarate) (PPF),^125^, ^126^ poly(lactic‐*co*‐glycolic) acid (PLGA),^18^ and PCL,^16^ have also been used in tarsal plate reconstruction in recent studies. Among them, only porous high‐density PE (Medpor®) has been used as an eyelid spacer for treating lower eyelid retraction in clinical practice.^123^ However, this material should be used sparingly in eyelid surgery, due to high postoperative complication rates, such as poor stability, implant exposure, abnormal skin contours, and unexplained pain.^127^ Other synthetic scaffolds are available and have been investigated for tarsal reconstruction in animal models. However, poor tissue tolerance impedes their clinical translation, and inflammatory tissue responses have been observed around the implants.^7^

The common advantages of these polymers are good biodegradability, rigidity, and moldability. However, compared with scaffolds derived from natural tissues, synthetic polymers have relatively low biocompatibility and usually have hydrophobic features because of the lack of natural cell recognition sites,^7^, ^128^ which limits their clinical translation. These limitations have prompted attempts to modify their biological characteristics by combining several differential biomaterials to make scaffolds with properties more closely resembling those of the natural tarsal plate. For example, with its excellent mechanical integrity and cytocompatibility, the biodegradable biopolymer PLA is widely used in the biomedical field. However, this material is hydrophobic, which is unfavorable for cell adhesion and growth.^128^ It has been considered a novel direction of tissue engineering to blend PLA with other biomaterials with better wettability, which could result in better tissue recovery. Yan et al.^12^ developed a PLA electrospun nanofibrous membrane scaffold for conjunctival repair, which was coated with cellulose nanofibrils to improve hydrophilicity, coated with silk peptide to promote conjunctival epithelial cell proliferation, and loaded with levofloxacin to prevent postoperative infection. By promoting the restoration of conjunctival structure and function, this scaffold yielded favorable therapeutic effects after transplantation in a rabbit conjunctival defect model.^12^ In addition, to improve the hydrophobicity and modulate the biomechanical property of pure PPF polymer, a degradable and mechanically adjustable scaffold was synthesized by copolymerizing PPF with 2‐hydroxyethyl methacrylate (HEMA), a widely used hydrophilic monomer. The PPF‐HEMA scaffold showed no cytotoxicity in vitro, elicited less inflammatory response and showed better tissue infiltration than the acellular dermal matrix control in tarsal plate reconstruction in rabbits.^125^ This copolymerized scaffold was then further modified by depositing a soft collagen/chitosan layer onto the PPF‐HEMA layer to fabricate a biomimetic biphasic scaffold. In a rabbit transconjunctival defect model, this biphasic scaffold allowed tissue to grow inward and maintained the contour of the eyelid, while the collagen/chitosan phase was able to support re‐epithelialization with functional palpebral conjunctiva.^126^

## 5 CELLULAR APPROACHES

Although numerous “smart” biomimetic biomaterials with the potential to guide tissue regeneration have been developed for posterior lamellar reconstruction,^12^, ^126^ biomaterial‐guided tissue regeneration extensively relies on the surrounding tissue microenvironment, which may not be suitable in some clinical scenarios, especially in cases of local inflammation, systemic autoimmune diseases, poor vascular beds, or massive or even complete loss of posterior lamellar tissue. Thus, acellular approaches are usually accompanied by fibrotic tissue formation, which can restore the mechanical support function of the eyelid but does not restore the secretory function and barely induces reepithelialization. Recently, progress has been made in the engineering of a cellular graft that can restore both the structural and functional properties of the posterior lamellar, especially lipid secretion and the smooth mucosal surface.

### 5.1 Tissue‐engineered tarsal plate equivalents

In 2019, Dai et al.^18^ engineered a composite construct consisting of PLGA sponge, bone marrow‐derived mesenchymal stem cells (BMSCs), plasmid DNA which encodes transforming growth factor‐β1, and fibrin gel. When the construct was grafted into the tarsal plate defects in a rabbit model, the defects showed both structural and functional reconstruction 8 weeks postimplantation, as demonstrated by sufficient ECM deposition and meibomian gland acinar unit formation within the implants.^18^ Although the origin of the meibomian glands in this study remains controversial, restoration of the lipid secretion function of the tarsus opened a new avenue in posterior lamellar tissue engineering. With the same aim of engineering a tarsal graft with lipid secretion function, Chen et al.^16^ developed biomimetic tarsal plate substitutes by coating a 3D‐bioprinted PCL scaffold with decellularized matrixes of adipose derived mesenchymal stem cells to improve hydrophilicity and cell adhesion properties of PCL‐based materials. In vitro studies revealed that this biomimetic scaffold had excellent cytocompatibility and supported SZ95 sebocyte proliferation. More importantly, 1 month after subcutaneous dorsal implantation into nude mice, the seeded SZ95 sebocytes showed good proliferation with structures similar to the meibomian gland acinar and an intact epithelial layer. Meanwhile, the seeded SZ95 sebocytes secreted abundant neutral lipids that were widely distributed within the scaffold. This study suggests that the lipid secretory function of tarsal plate substitutes could be restored by seeding lipid‐secreting cells onto bioscaffolds. However, orthotopic tarsal defect reconstruction studies are required to further test the secretory function of the regenerated meibomian glands.

### 5.2 Tissue‐engineered conjunctival equivalents

As far as we know, no study has investigated cellular approaches to repair palpebral conjunctival defects so far. Notably, numerous studies on bulbar conjunctival tissue engineering have demonstrated encouraging results in the promotion of reepithelialization and reduction of scarring in both animal models^17^, ^32^, ^120^ and clinical trials^59^, ^129^ (Table 1). Considering that the palpebral conjunctiva has structures and functions similar to those of the bulbar conjunctiva,^30^ strategies for engineering bulbar conjunctival equivalents are translatable for engineering palpebral conjunctiva equivalents.

**TABLE 1:** Cellular approaches for conjunctival reconstruction.

Study	Seed cells	Scaffold	Highlight in vitro results	Highlight in vivo results
Conjunctival source				
Wu et al. (2020)^32^	Human and rabbit total, p75^−^, p75^+^, and p75^++^ CjECs	AM	‐Human p75^++^ CjECs showed the strongest proliferation potential. ‐Human p75^++^ CjECs formed the thickest conjunctival epithelium with 8–10 cell layers and the highest density of MUC5AC^+^ cells.	‐Large conjunctiva defect model in rabbits. ‐Conjunctiva epithelium engineered from rabbit p75^++^ CjECs achieved more complete reepithelization and more goblet cells than that engineered from control cells.
Hong et al. (2018)^133^	Human CjECs	PLGA membrane, PET membrane	‐Human CjECs formed epithelium with two to three cell layers in both groups. ‐PET group showed higher MUC5AC expression, while PLGA group showed higher CK19 and Ki67 expression.	‐Corneal defect model in rabbits. ‐PLGA‐based epithelial sheets achieved a faster and more complete reepithelization but without goblet cells compared to the PET scaffolds.
Witt et al. (2020)^17^	Human CEx, CjECs	PDC	‐CEx cultured on PDC formed a thicker epithelium with MUC5AC^+^ and PAS^+^ cells. ‐No goblet cells were observed in the cultured CjECs.	‐Conjunctival defect in rabbits. ‐PDC + CEx constructs showed preserved progenitor cell properties and higher goblet cell numbers than control groups.
Bandeira et al. (2019)^84^	Human CEx	uHAM	‐CEx cultured on uHAM formed multilayered stratified epithelium with MUC5AC^+^ cells after 3–4 weeks of expansion.	‐Clinical study on ocular surface reconstruction. ‐uHAM + CEx constructs repaired large epithelial defects without lesion recurrence in two patients throughout the follow‐up.
Nonconjunctival mucosal source				
Komai et al. (2021)^15^	Human oral mucosal explant	AM	/	‐Retrospective cohort study in fornix reconstruction involved 16 eyes of 15 patients. ‐Mean follow‐up was 102.1 ± 46 months. −5‐year overall fornix‐reconstruction success probability was 79.6% ± 10.7%, and success probability for thermal/chemical injury and OCP was 100% and 53.3% ± 24.8%, respectively.
Wang et al. (2019)^129^	Human limbal stem cells	AM	/	‐Retrospective case series in conjunctival sac reconstruction involved 36 eyes of 36 patients. ‐Mean follow‐up was 17.4 ± 3.9 months. ‐Symblepharon was completely relieved in 30 eyes 3 months after surgery. ‐No symblepharon recurrence during the 3‐month follow‐up period.
Kobayashi et al. (2015)^130^	Human nasal, oral, corneal, conjunctival mucosal cells	AM	‐All cell types formed epithelium with four to five layers of CK3^+^ and CK13^+^ cells. ‐MUC5AC^+^ cells were only detected in cultured nasal mucosal cells.	‐Conjunctival defect model in rabbits. ‐Transplanted CEMNS were well adopted to conjunctival defects and contained goblet cells at Day 14.
Other epithelial source				
Yang et al. (2015)^141^	Human amniotic epithelial cells	Decellularized AM	‐Amniotic epithelial cells that was induced with decellularized conjunctival matrix for 5 days expressed CK4, CK13, and MUC5AC. ‐Differentiated amniotic epithelial cells cultured on decellularized AM for 14 days formed four to five cell layers.	‐Conjunctival defect model in rabbits. ‐Engineered conjunctiva was covered with two to five layers of cells and contained MUC5AC^+^ cells, while fresh AM only had a single layer of epithelia.
Lu et al. (2011)^164^	Monkey epidermal progenitor cells	Denuded AM	‐Epidermal progenitor cells cultured on AM formed a multilayered epithelium without MUC5AC^+^ cells.	‐Conjunctival defect model in in rhesus monkey. ‐Autologous cultivated epidermal progenitor cells formed several layers of epithelial cells with similar appearance to those of normal conjunctival epithelium. ‐No goblet cells were detected in the sheets.

Abbreviation: AM, amniotic membrane; CEMNS, cultured epithelial mucosal nasal sheet; CEx, conjunctival explant; CjECs, conjunctival epithelial cells; OCP, ocular cicatricial pemphigoid; PAS, periodic acid Schiff; PDC, porcine decellularized conjunctiva; PET, polyester; PLGA, poly(lactic‐*co*‐glycolic) acid; uHAM, urea‐de‐epithelialized human amniotic membrane.

To meet the needs of conjunctival repair, a tissue‐engineered conjunctival epithelial substitute should have good surgical manipulability, excellent biocompatibility, and low inflammatory properties. Histologically, it needs to be a stratified epithelium composed of squamous epithelial cells, goblet cells, and stem/progenitor cells. By following the principles of tissue engineering, a conjunctival equivalent with a multilayered stratified epithelium and individual interspersed goblet cells can be developed in vitro by cultivating conjunctival explants on a matrix that has supportive niche features to facilitate adhesion, proliferation, as well as differentiation of epithelial and goblet cells under defined conditions. Various biomaterials for direct conjunctival replacement, as mentioned above, ranging from decellularized matrixes to artificial synthetic polymers, could possibly be adapted for conjunctival equivalent engineering, such as decellularized amniotic membranes,^32^, ^130^ decellularized porcine conjunctiva,^17^ decellularized porcine pericardium,^10^ fibrin,^131^, ^132^ collagen,^112^, ^113^ PLGA,^133^ PCL,^118^, ^134^ polyester,^133^ and 3D‐printed membranes based on a blend of previously described biomaterials.^81^ These biomaterials act as carrier scaffolds of epithelial cells during in vitro culture. However, as mentioned earlier, the main problems with current synthetic scaffolds include difficult surgical manageability, poor biocompatibility, and inadequate biomechanical strength in vivo.

A wide range of cell types has been used for developing bioengineered conjunctival equivalents. Conjunctival epithelial cells are the most frequently used cells for conjunctival equivalent engineering. Conjunctival epithelial cells cultured on plastic compressed collagen gels,^112^ and amniotic membranes^84^ retained their characteristic cuboidal shape with mucin expression, indicating that these substrata may influence the differentiation of goblet cells. Multilayered conjunctival epithelial cell organization was achieved by culturing human conjunctival epithelial cells on electrospun bioscaffolds consisting of PCL and decellularized tissue matrix.^134^ Furthermore, goblet cell repopulation, a hallmark of conjunctival epithelium, was observed in bioengineered conjunctival equivalents developed from conjunctival epithelial cells^120^, ^133^, ^135^ and conjunctival stem cells.^136^ However, the use of conjunctival epithelial cells and conjunctival stem cells requires expansion in vitro, which in turn requires a stem cell niche‐like matrix or feeder cell support before cultivation on a presumptive substrate.^137^, ^138^ Furthermore, the isolation, expansion, and preservation of conjunctival epithelial cells as well as conjunctival stem cells in vitro require xenobiotic components that limit their clinical application. Thus, engineering stable, stratified, and goblet cell‐rich constructs can be achieved by explant culture of small conjunctival samples on decellularized conjunctiva^17^, ^135^ or amniotic membrane.^139^, ^140^ Such conjunctival explants can form a thicker epithelium with more MUC5AC‐positive goblet cells on decellularized porcine conjunctiva than conjunctival epithelial cells.^17^ A possible interpretation of the superiority of conjunctival explants over conjunctival epithelial cells is that the conjunctival stem cell populations within the conjunctival explants could maintain their undifferentiated state longer as they are located closer to their niche environment.

Conjunctival epithelial equivalents containing goblet cells have been engineered from nonconjunctival cell sources, such as nasal mucosal epithelial cells,^130^ which harbor goblet cells, and amniotic epithelial cells, which are able to differentiate into conjunctival epithelial cells and goblet cells under specific conditions.^141^ For patients with a large conjunctival deficiency or scarring, oral mucosal epithelial cells are considered as an alternative source of seed cells for conjunctival epithelial equivalent engineering strategies and have been applied in clinical practice in the form of a cultivated oral mucosal epithelium transplantation technique for ocular surface reconstruction^59^, ^61^ and forniceal reconstruction.^15^ Although promising results have been achieved in terms of high rates of successful forniceal reconstruction, the engineered oral epithelium lacks goblet cells, which limits its usefulness for conjunctival reconstruction.

## 6 DISCUSSION AND FUTURE PERSPECTIVES

### 6.1 Future avenues: From reparative to regenerative surgery

Although the injured conjunctiva is able to reepithelialize spontaneously, the accompanying fibrosis can lead to forniceal shortening and even ankyloblepharon, followed by corneal diseases and/or dry eye diseases, which may lead to ultimate vision loss.

Numerous techniques have been developed for posterior lamellar reconstruction over the past decades; however, the eyelid reconstruction techniques currently available in clinical practice are essentially replacement strategies. When this type of strategy is implemented, autografts or cell‐free biomaterials are implanted into the defect area to provide mechanical support. These biomaterials are easily harvested and relatively inexpensive, but high rates of complications have also been reported, such as graft contraction and shrinkage,^102^ graft exposure,^98^ and cyst formation.^14^ Most importantly, due to the lack of posterior lamellar cellular components, these grafts barely provide a smooth epithelialized surface and restore secretory function. Although some studies have reported that implanted decellularized matrixes could support cell growth both in vitro^84^ and in vivo,^85^ concomitant fibrosis rather than complete reepithelization was observed on the surface of the biomaterials upon implantation in animal models.^10^, ^109^ Cellular approaches aim to restore the full function of the posterior lamella by implanting a tissue‐engineered tarsal or conjunctival equivalent into the defect area, which can be referred to as “regenerative surgery” and represents a future direction in this field. For example, by seeding immortalized human SZ95 sebocytes onto a scaffold, a tarsal plate substitute capable of secreting neutral lipids both in vitro and in vivo was successfully engineered.^16^ Moreover, by culturing conjunctival epithelial cells on vitrified collagen membrane, conjunctival equivalents that could greatly promote goblet cell repopulation as well as minimize fibrosis and wound contracture was successfully developed.^113^ These results, at least partially, reproduced the secretory function of the human eyelid. However, the field remains in its infancy, and extensive investigation is required in the future.

### 6.2 Future challenges to functional eyelid reconstruction

#### 6.2.1 Bridging the gap with 3D bioprinting technology

The human tarsal plate has three key characteristics: a semilunar shape, a thickness of 1 mm, and a certain degree of mechanical strength. Thus, tarsal plate substitutes should possess similar features to fit clinical conditions. However, neither native nor bioengineered tarsal plate substitutes that are currently available fully demonstrate these characteristics. For example, auricular cartilage grafts are thin, relatively abundant, and mechanically similar to the native tarsus and thus have been widely used in clinical conditions and have provided satisfactory esthetics and appropriate support for the eyelid without apparent shrinkage or absorption in several studies.^2^, ^8^ However, the contour of native auricular cartilage does not fit the contour of the globe, resulting in corneal irritation.^74^ Porous Medpor® is a biocompatible and rigid material that can be easily molded into an ultrathin and curved eyelid spacer for the treatment of eyelid retraction.^123^, ^142^ However, porous Medpor® eyelid spacers show low tissue tolerance, with an implant exposure rate as high as 76.5%,^143^ which significantly hinders their widespread use.

The development of 3D bioprinting technology has the potential to bridge this gap. This technology involves depositing cell‐loaded biomaterials layer by layer in predetermined architecture to produce artificial multicellular tissues and/or organs with biomimetic microstructures.^144^, ^145^ Recently, bioprinted tracheal,^146^, ^147^ human nasal cartilage,^148^, ^149^ and long bone^150^, ^151^, ^152^ constructs have been successfully engineered. These bioprinted constructs exhibit considerable mechanical strength for maintaining specific geometric shapes or bearing loads, contain tissue‐specific cells for generating functional tissues *in vivo*, and even have well‐designed microstructures to serve special functions, such as promoting angiogenesis^153^, ^154^ and guiding tissue regeneration.^155^, ^156^, ^157^ 3D cell bioprinting technology allows personalized designs, precise preparation procedures, and on‐demand creation within a short time frame,^144^, ^158^ making the fabrication of shape‐customized and mechanically strengthened tarsal substitutes possible. In addition, 3D bioprinting technology has been used in conjunctival equivalent engineering. For example, 3D‐bioprinted gelatin‐based membranes have been reported to offer necessary mechanical and physical properties required for effective conjunctival defect reconstruction.^81^ Compared to the amniotic membrane, the 3D‐printed membrane had a higher density of goblet cells on the regenerated epithelium and had a more predictable degradation pattern, less scar formation, and minor inflammatory responses in vivo.^81^ Another study demonstrated the successful application of bioprinted hydrogel microconstructs loaded with rabbit conjunctival stem cells for conjunctival regeneration.^159^ The bioprinted microconstructs favorably mimicked the biochemical and biomechanical properties of the native stem cell niche matrix of the conjunctiva. Therefore, they were capable of maintaining their stem cell phenotype and effectively supporting conjunctival stem cell growth and differentiation into squamous epithelial cells as well as goblet cells. With the use of 3D bioprinting, posterior lamellar substitutes could be successfully fabricated with the required mechanical properties and composite structures, and various types of seed cells can be fully expected in the future.

#### 6.2.2 Focusing on secretory function

The restoration of secretory function remains another great challenge in eyelid reconstruction. Goblet cells within the conjunctiva and meibomian glands within the tarsus are responsible for secreting lipids and mucins, respectively, which participate in tear film formation and maintain ocular surface homeostasis.^160^, ^161^ Engineering tarsal plates and conjunctival equivalents able to secrete lipids and/or mucins presents a potential strategy to solve this problem. Stem cells involved in the development of meibomian glands have not been well demonstrated thus far, posing an obstacle to meibomian gland regeneration. Recently, a stem/progenitor cell population expressing Krox20, a zinc finger transcription factor, was identified as a crucial driver of the development and homeostasis of the meibomian gland,^162^ shedding light on the identification of meibomian gland stem cells and the subsequent construction of meibomian gland substitutes. In addition to regenerating the meibomian glands, engineering tarsal equivalents by directly seeding lipid‐secreting sebocytes onto scaffolds presents another feasible approach.^16^ However, sebocytes are terminally differentiated cells with limited self‐renewal capability, and the amounts and types of secreted lipids and the durability of the lipid secretion function still require further in‐depth investigation. Although goblet cells account for only 5%–10% of cells of the conjunctival epithelium, they play an irreplaceable role in lubrication, immunomodulation, and ocular surface homeostasis.^31^ Conjunctival stem cells are able to differentiate into goblet cells and conjunctival epithelial cells under physiological conditions, but this process often fails to reappear in tissue‐engineered conjunctival equivalents.^30^, ^163^ For example, Witt et al. preexpanded human conjunctival epithelial cells on porcine decellularized conjunctiva for 14 days in vitro, and a stratified epithelium with an average thickness of 15.15 μm was formed, but no goblet cells were found in the epithelium.^17^ Methods to increase goblet cell numbers have mainly focused on directing resident stem cells toward goblet cell differentiation.^139^

#### 6.2.3 Bioengineering a bilayered construct for transconjunctival reconstruction

Anatomically, the native posterior lamella of the eyelid is a bilayered structure, with each layer exhibiting unique structures and functions. In clinical conditions, posterior lamellar defects are often accompanied by conjunctival insufficiency. Thus, the ideal bioengineered posterior lamellar substitute should also be a bilayered hybrid, with one lamella providing mechanical strength and the other providing a smooth epithelial surface. However, most recent advances in posterior lamellar reconstruction meet one of the three requirements by using a single tarsal or conjunctival substitute,^6^, ^11^, ^66^ undoubtably achieving inferior reconstructive outcomes. Recently, a biphasic scaffold composed of a hard PPF–HEMA layer and a soft collagen/chitosan layer was developed to mimic the bilayered structure of the native posterior lamella.^126^ Two weeks after implantation into tarsoconjunctival defects in a rabbit model, the histological findings demonstrated that the hard, porous PPF–HEMA layer supported the shape of the eyelid and allowed the ingrowth of surrounding tissue, while the soft collagen/chitosan sponge layer promoted cell migration and conjunctival regeneration,^126^ suggesting the potential of this construct for tarsoconjunctival repair. However, similar to other acellular approaches, concerns regarding the efficiency of restoring secretory function and a smooth epithelial lining due to the lack of cellular components remain with this approach. Unfortunately, to our knowledge, no bilayered cellular substitutes for posterior lamellar reconstruction have been reported in the literature. By using integrated techniques, such as 3D bioprinting and advanced biomaterials, engineering a bilayered cellular substitute for posterior lamellar reconstruction is feasible and may solve the remaining problems.

## 7 CONCLUSION

In conclusion, posterior lamellar reconstruction remains a great challenge because of the delicate bilayer structure and complicated functions of the eyelid. An ideal posterior lamellar substitute should exhibit three features: sufficient mechanical strength, a smooth epithelial surface, and lipid secretion function. However, currently available clinical autografts and techniques for posterior lamellar reconstruction do not fully meet these requirements, which has stimulated the exploration of acellular and cellular bioengineered substitutes for posterior lamellar reconstruction. Acellular approaches via replacing defective posterior lamellar tissue with biomimetic biomaterials have demonstrated promising outcomes in both experimental studies and clinical conditions. Cellular approaches aim to promote posterior lamellar tissue regeneration by implanting living tissue‐engineered grafts, but these methods are still in their infancy. Future studies on biphasic scaffold design and the extensive use of 3D printing technology will facilitate the design of successful tissue‐engineered bilayer cellular substitutes with secretory functions for eyelid reconstruction.

## AUTHOR CONTRIBUTIONS

**Yuxin Yan:** Conceptualization (equal); data curation (equal); investigation (equal); methodology (equal); resources (equal); writing – original draft (equal). **Qiumei Ji:** Conceptualization (equal); data curation (equal); investigation (equal); methodology (equal); visualization (equal); writing – review and editing (equal). **Rao Fu:** Data curation (equal); formal analysis (equal); investigation (equal). **Chuanqi Liu:** Data curation (equal); investigation (equal); methodology (equal). **Jing Yang:** Data curation (equal); investigation (equal); methodology (equal). **Xiya Yin:** Data curation (equal); investigation (equal); methodology (equal). **Qingfeng Li:** Conceptualization (equal); data curation (equal); project administration (equal); resources (equal); writing – review and editing (equal). **Ru‐Lin Huang:** Conceptualization (equal); data curation (equal); project administration (equal); resources (equal); funding acquisition (equal); writing‐ review and editing (equal).

## CONFLICT OF INTEREST STATEMENT

The authors declare no conflict of interest.

### PEER REVIEW

The peer review history for this article is available at https://publons.com/publon/10.1002/btm2.10497.

## Data Availability

The data that support the findings of this study are available from the corresponding author upon reasonable request.
